# IL-17 Promotes Angiogenic Factors IL-6, IL-8, and Vegf Production via Stat1 in Lung Adenocarcinoma

**DOI:** 10.1038/srep36551

**Published:** 2016-11-07

**Authors:** Qi Huang, limin Duan, Xin Qian, Jinshuo Fan, Zhilei Lv, Xiuxiu Zhang, Jieli Han, Feng Wu, Mengfei Guo, Guorong Hu, Jiao Du, Caiyun Chen, Yang Jin

**Affiliations:** 1 Department of Respiratory and Critical Care Medicine, Key Laboratory of Pulmonary Diseases of Health Ministry, Union Hospital, Tongji Medical College, Huazhong University of Science and Technology, 1277 Jiefang Avenue, Wuhan, 430022, China; 2 Department of Respiratory Medicine, Taihe Hospital, Hubei University of Medicine, No. 32, South Renmin Road, Shiyan, Hubei, 442000, P.R. China; 3 Zhongshan Hospital, Xiamen University, 201-209 Hubin Road, Xiamen, Fujian, 361004, P.R. China; 4 Department of Respiratory Medicine,the First Hospital of Xi′an City, Xi′an, Shanxi, 710002, P.R. China

## Abstract

Inflammation and angiogenesis are two hallmarks of carcinoma. The proinflammatory cytokine interleukin-17 (IL-17) facilitates angiogenesis in lung cancer; however, the underlying mechanism is not fully understood. In this study, tumour microvessel density (MVD) was positively associated with IL-17, interleukin-6 (IL-6), interleukin-8 (IL-8), and vascular endothelial cell growth factor (VEGF) expression in human lung adenocarcinoma tissues, and it was increased in tumour tissues of A549-IL-17 cell-bearing nude mice. Importantly, positive correlations were also detected between IL-17 expression and IL-6, IL-8 and VEGF expression in human lung adenocarcinoma tissues. Furthermore, IL-6, IL-8 and VEGF production, as well as STAT1 phosphorylation, were increased in tumour tissues of A549-IL-17 cell-bearing nude mice *in vivo* and in A549 and H292 cells following IL-17 stimulation *in vitro*. In addition, STAT1 knockdown using an inhibitor and siRNA attenuated the IL-17-mediated increases in IL-6, IL-8 and VEGF expression in A549 and H292 cells. In conclusion, IL-17 may promote the production of the angiogenic inducers IL-6, IL-8 and VEGF via STAT1 signalling in lung adenocarcinoma.

Cancer is a leading cause of death in both more and less economically developed countries^1^. Among males, lung cancer is the leading cause of cancer-related death in these countries, and among females, it is the leading cause of cancer-related death in more developed countries and the second leading cause in less developed countries^1^,^2^. Non-small-cell lung cancer (NSCLC) accounts for approximately 80% of lung cancers, and adenocarcinoma is its most common histological type. Despite the substantial advances that have been achieved, the overall 5-year survival rate of lung cancer patients is still approximately 15%^3^ because of metastasis and recurrence.

Angiogenesis is a major hallmark of malignancy^4^,^5^ and can be evaluated by tumour microvessel density (MVD), as demonstrated widely by immunocytochemical staining for CD31 and CD34 in tumours^6^, including lung cancer^7^,^8^. Angiogenesis is coordinated by several types of molecules^5^, such as chemokines and cytokines. Inflammation is another major hallmark of malignancy^4^,^5^, including lung cancer^9^,^10^. Interleukin-17 (IL-17), a newly identified proinflammatory cytokine that is mainly produced by Th17 cells^11^, has been widely investigated in many human solid tumours^12^, including in lung cancer. In lung cancer, IL-17 triggers tumour progression, mainly due to its proangiogenic properties by stimulating the production of angiogenic factors. Many cytokines, such as interleukin-6 (IL-6)^13^, interleukin-8 (IL-8)^14^,^15^ and vascular endothelial cell growth factor (VEGF)^16^, are well-known angiogenic inducers, and IL-17 induces these cytokines in lung cancer. However, the mechanism by which IL-17 mediates the secretion of these angiogenic factors remains unclear.

IL-17 communicates with Jak-Stat family signalling, particularly STAT3, in many diseases^17^. Several reports have revealed that STAT3 is involved in IL-17-induced VEGF production in malignant tumours^18^,^19^,^20^, including lung cancer^15^, and one study^7^ has demonstrated that IL-17 influences IL-6, IL-8 and VEGF expression in lung cancer cell lines via an unknown mechanism. However, the role of Jak-Stat family signalling, such as STAT1 signalling, in the IL-17-mediated regulation of IL-6, IL-8 and VEGF in lung adenocarcinoma remains unknown.

In the present study, we (i) measured IL-17, MVD, IL-6, IL-8 and VEGF expression and examined their associations in human lung adenocarcinoma tissues; (ii) measured MVD, IL-6, IL-8, VEGF and STAT1 expression in A549-IL-17 cell-bearing nude mice; and (iii) determined the effects of a STAT1 inhibitor and siRNA on IL-17-induced IL-6, IL-8 and VEGF expression in A549 and H292 cells *in vitro*. Our results will provide a better understanding of the communication among IL-17, IL-6, IL-8, and VEGF as well as new insights into the targeting of inflammation and angiogenesis for the treatment of lung cancer.

## Materials and Methods

### Clinical samples

Tumor and the corresponding peritumor tissues of 60 patients with lung adenocarcinoma were obtained from the Department of Thoracic Surgery, Union Hospital, Tongji Medical College, Huazhong University of Science and Technology from 2012 and 2016. All patients did not receive any other treatments before surgery and provided written informed consents. According to the 7th edition of the National Comprehensive Cancer Network (NCCN) guidelines version 1. 2016, the TNM classification were proved to be lung adenocarcinoma by Department of Pathology, Union Hospital, Tongji Medical College, Huazhong University of Science and Technology. All experiments were performed in accordance with relevant guidelines and regulations and the study was approved by the ethical committee of Union Hospital, Tongji Medical College, Huazhong University of Science and Technology ([2010] IEC (S202)).

### Cell cultures

The human lung adenocarcinoma cell lines A549 (ATCC #CCL-185) and H1299 (ATCC #CRL-1848™) were purchased from ATCC (Manassas, VA, USA) and cultured as previously described^21^. Recombinant human IL-17 was purchased from PeproTech (Rocky Hill, NJ). The STAT1 inhibitor (fludarabine) was obtained from HISUN Pharmaceutical Co. Ltd. (Zhejiang, China).

### Tumour model

For plasmid construction, human IL-17 complementary DNA (cDNA) was purchased from Gene Chem (Shanghai, China). A recombinant vector and mock plasmid were transfected into A549 cells using Lipofectamine 2000 (Invitrogen, Carlsbad, CA). The stably expressing cell line and mock-transfected cells were selected with G418 (1000 μg/ml, Sigma). The IL-17 mRNA and protein levels in the A549 cells (A549-IL-17 and A549-Neo) were verified by immunofluorescence and real-time PCR (the data are shown in the Supplementary Figure 1).

Male nude mice were obtained from Beijing HFK Bio-Technology (No. 11401300002023). These mice were kept in laminar flow cabinets under specific pathogen-free conditions and were acclimated to the environment for 1–2 weeks before the experiments were performed. All of the animal experimental procedures were approved by the Animal Care and Use Committee of Tongji Medical College. We have ensured that all of the animal experimental procedures were performed and approved in accordance with the relevant guidelines and regulations by the Animal Care and Use Committee of Tongji Medical College.

Male nude mice aged approximately 4–6 weeks were randomly allocated into two groups. The mice in one group were subcutaneously injected with A549-IL-17 cells (1 × 10^6^ cells/mouse), and those in the other group were injected with A549-Neo cells (1 × 10^6^ cells/mouse). The weights and tumour volumes (TVs) were measured for all mice every three days after their subcutaneous tumours became detectable. The TVs were calculated according to the following formula: TV (cm^3^) = a × b^2^/2, where a and b are the longest and shortest diameters, respectively. All of the mice were sacrificed after receiving intraperitoneal anaesthesia with pentobarbital sodium (40 mg/kg in a 1% saline solution), which was administered at 36 days after injection to the nude mice. Fresh tumour tissues were fixed with paraformaldehyde before being embedded in paraffin.

### Immunohistochemistry (IHC)

The procedure for IHC staining of tumour tissues from humans and mice was described previously^21^, and the following antibodies were used for this analysis: anti-human IL-17 and anti-human VEGF (1:100; Abcam, Cambridge, UK), anti-human IL-6 and anti-human IL-8 (1:100; Bioss, Beijing, China), and anti-CD31 (1:1; Maixin, Fujian, China). For MVD counting, MVD was determined based on the average of CD31 IHC staining positive cells counting. Cell membrane of vascular endothelial cells and (or) cytoplasm showed brown staining. MVD assessment and counting methods: low magnification visual wild find three select microvessel most abundant of the “hot spots”, and then high power field (x400) Counted, the average value as the specimen MVD (/field) were recorded by experienced observers in Department of Pathology of our hospital who were blind to the details of the patients. And IL-17, IL-6, IL8. Images were analysed as previously described^22^.

### RNA interference

Human STAT1-siRNA (sc-44123) and human control-siRNA (sc-siRNA and sc-37007) were obtained from Santa Cruz Biotechnology. siRNAs were transfected into A549 and H292 cells using an siRNA Reagent System (sc-29528, Santa Cruz Biotechnology) at a final concentration of 80 nM for 48 h according to the manufacturer’s protocol.

### Western blotting (WB)

A549 and H292 cells (1 × 10^6^ cells/well) were seeded in six-well plates overnight and were then exposed to the different treatments at the indicated time points. Cell lysates and tumour tissue homogenates were separated by SDS–PAGE and then transferred onto PVDF membranes (Millipore, USA). Next, WB was performed as previously described^23^. The following specific primary antibodies were used: anti-phospho-STAT1 (1:1000, Tyr701, CST#9167), anti-STAT1 (1:1000, Tyr701, CST#9167), anti-IL-6 (1:1000, 21865-1-AP), anti-IL-8 (1:1000, sc-1265; Santa Cruz Biotechnology), anti-VEGF (1:1000, BA0407; Boster, China), and anti-GAPDH (1:5000, BL1039; Wuhan, China).

### Quantitative real-time PCR (qRT-PCR)

Total RNA was extracted from cells and tissues, and the extracted RNA was reverse transcribed and amplified by qRT-PCR as previously described^22^. The mRNA levels of the target genes were normalized to GAPDH. The sequences of the primers used for PCR are presented in Supplementary Table 1 (TS1).

### Enzyme-linked immunosorbent assay (ELISA)

A549 and H292 cells (1 × 10^5^ cells/well) were exposed to the different treatments for 48 h, and then the IL-6, IL-8 and VEGF protein levels in the culture supernatants were measured using ELISA kits according to the manufacturer’s protocols (all kits were purchased from R&D Systems, Minneapolis, MN).

### Statistics

The results are presented as the mean ± SEM. Differences were evaluated for significance using the t-test or one-way analysis of variance. Correlations were assessed in human lung adenocarcinoma tissue samples. Analyses were performed using SPSS statistical software version 16.0 (Chicago, IL), and a two-tailed p < 0.05 was considered to indicate statistical significance.

## Results

### MVD is positively related to IL-17, IL-6, IL-8, and VEGF expression in human lung adenocarcinoma

**Figure 1:**
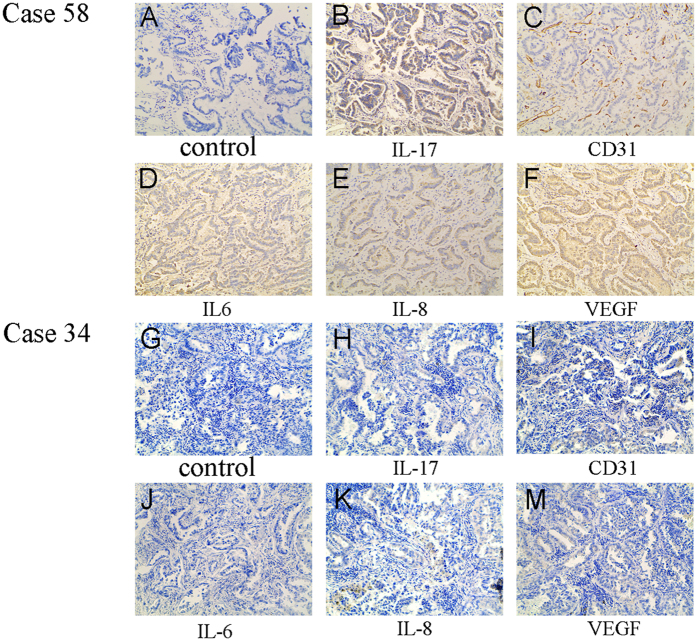
Expression of CD31, IL-17, IL-6, IL-8, and VEGF protein in human lung adenocarcinoma tissues. Immunohistochemical determination of CD31, IL-17, IL-6, IL-8, VEGF protein in 30 patients with lung adenocarcinoma. (**A**–**F**) High CD31, IL-17, IL-6, IL-8, VEGF protein expression were presented in tumor tissue of case 58. (**G**–**L**) Low CD31, IL-17, IL-6, IL-8, VEGF protein expression were showed in tumor tissue of case 34. No staining was observed when an isotype-matched control mAb (**A**,**G**) was used (magnification, × 200).

**Figure 2:**
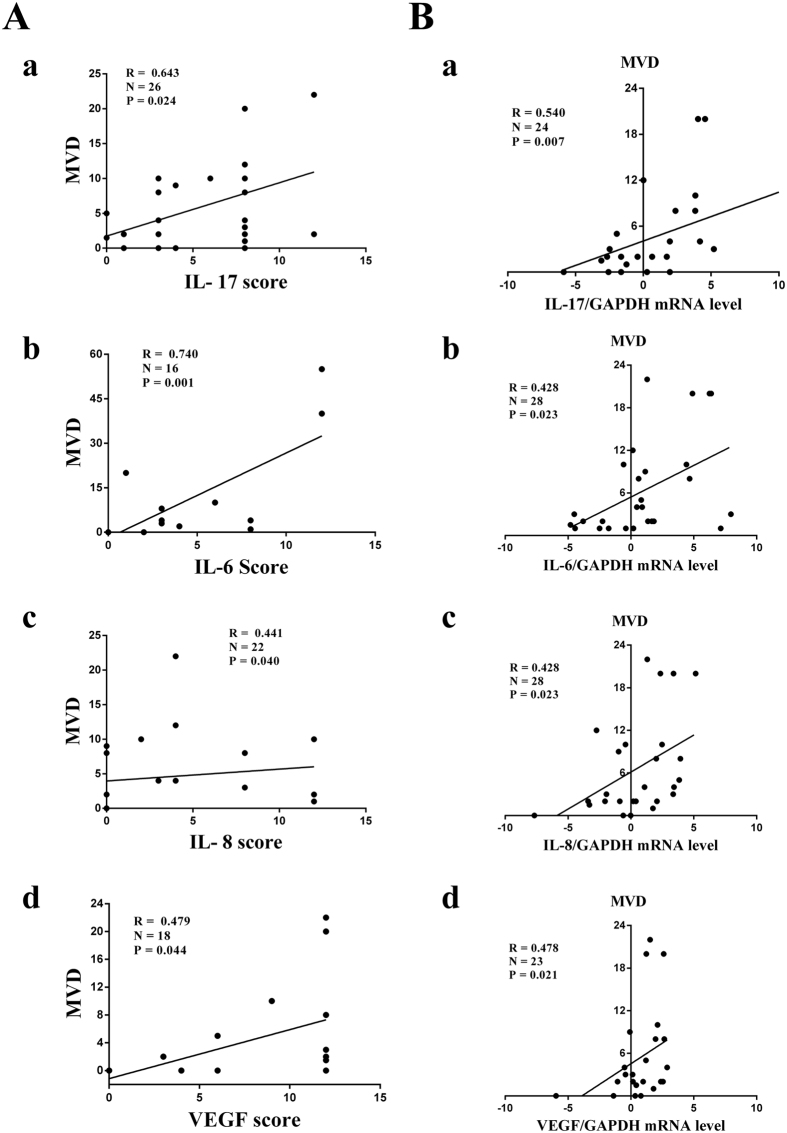
Correlation between MVD and IL-17, IL-6, IL-8, VEGF in human lung adenocarcinoma tissues. IL-17, IL-6, IL-8, VEGF mRNA and protein and CD31 protein levels were determined in human lung adenocarcinoma tissues by qRT-PCR or IHC, respectively. **(A)** Spearman’s correlation analysis was performed to analyse the correlation between IL-17 (a), IL-6 (b), IL-8 (c), VEGF (d) protein expression and tumour microvessel density (MVD) by CD31 staining in tumor tissues with human lung adenocarcinoma. **(B)** Spearman’s correlation analysis was performed to analyse the correlation between IL-17 (a), IL-6 (b), IL-8 (c), VEGF (d) expression and tumour microvessel density by CD31 staining in 28 tissues with human lung adenocarcinoma. mRNA expression levels were calculated using the −ΔΔCt method, and target gene expression was normalized to the GAPDH housekeeping gene.

Angiogenesis is coordinated by several types of molecules^5^, such as chemokines and cytokines. In our study, the relationship between IL-17, IL-6, IL-8, and VEGF and MVD determined by CD31 staining in human lung adenocarcinoma tissues was explored by qRT-PCR and IHC. Our results revealed that MVD was positively associated with IL-17, IL-6, IL-8, and VEGF protein expression in human lung adenocarcinoma tissues (Fig. 1A–J and Fig. 2A,B). Furthermore, MVD was also positively correlated with the IL-17, IL-6, IL-8, and VEGF mRNA level (Fig. 2E–H), suggesting that IL-17, IL-6, IL-8, and VEGF may be involved in angiogenesis in human lung adenocarcinoma.

### IL-17 is positively related to IL-6, IL-8, and VEGF expression in human lung adenocarcinoma

**Figure 3:**
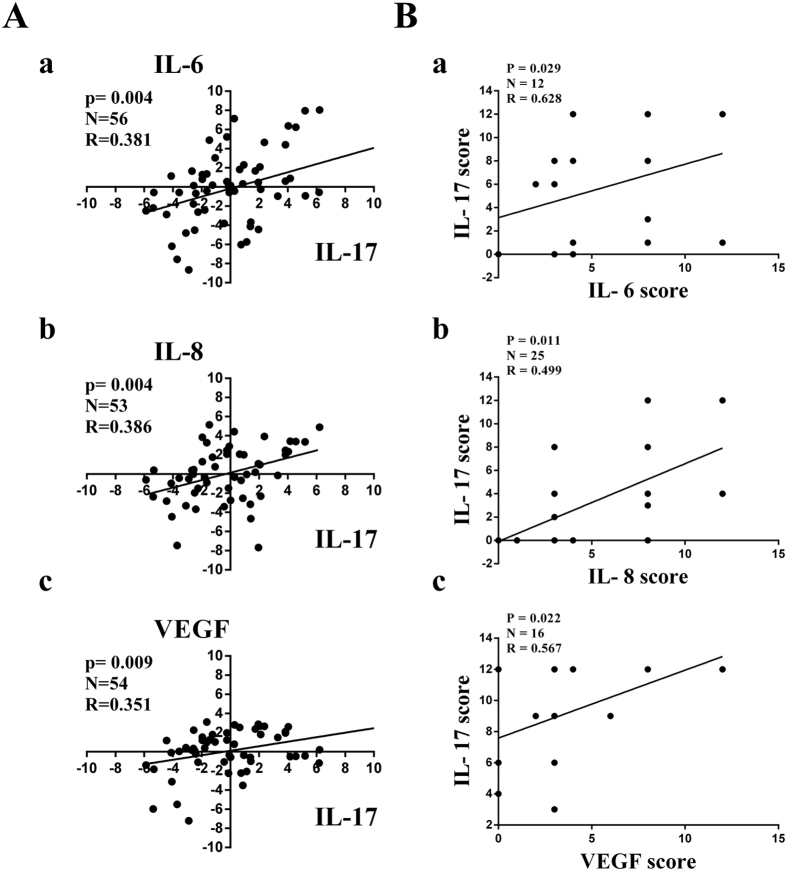
Correlation between IL-17 and IL-6, IL-8, VEGF in human lung adenocarcinoma tissues. IL-17, IL-6, IL-8, VEGF mRNA and protein protein levels were determined in human lung adenocarcinoma tissues by qRT-PCR or IHC, respectively. **(A)** Spearman’s correlation analysis was performed to analyse the correlation between IL-17 protein expression and IL-6 (a), IL-8 (b), VEGF (c) protein in tumour tissues of patients with lung adenocarcinoma. **(B)** Pearson’s correlation analysis was used to analyse the relationship between IL-17 mRNA expression and IL-6 (a), IL-8 (b), VEGF (c) mRNA expression in tumour tissues of patients with lung adenocarcinoma.. mRNA expression levels were calculated using the −ΔΔCt method, and target gene expression was normalized to the GAPDH housekeeping gene.

Next, we evaluated the association of IL-17 expression with IL-6, IL-8, and VEGF expression in lung adenocarcinoma tissues by qRT-PCR and IHC. Our findings demonstrated that the mRNA and protein levels of IL-6, IL-8 and VEGF were positively correlated with those of IL-17 mRNA and protein, respectively (Fig. 3A,B), indicating that IL-17 may induce angiogenesis by regulating the production of angiogenic factors IL-6, IL-8, and VEGF expression in human lung adenocarcinoma.

### IL-17 facilitates IL-6, IL-8, and VEGF production in lung adenocarcinoma cells *in vitro*

**Figure 4:**
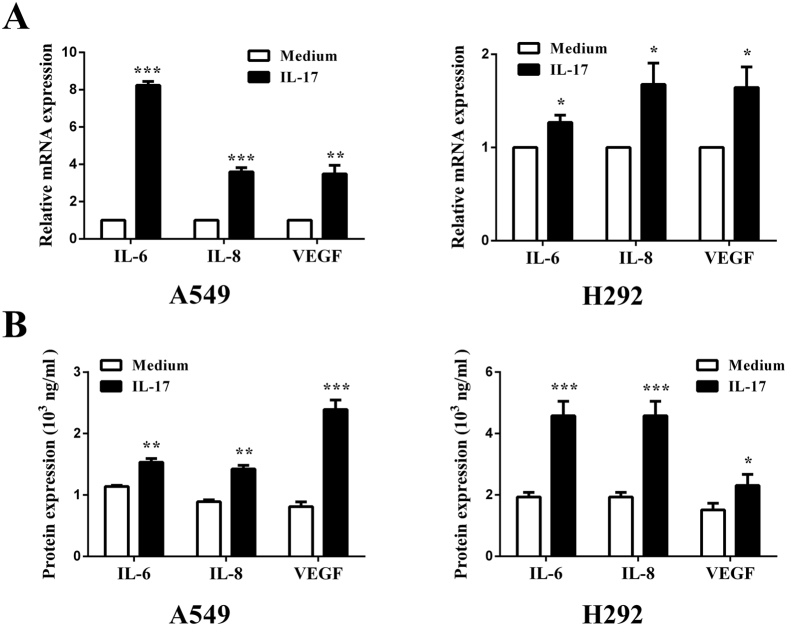
The effect of IL-17 on IL-6, IL-8, VEGF expression in human lung adenocarcinoma cells *in vitro*. A549/H292 cells were incubated with IL-17 or IL-17 (100 ng/ml) for 6 or 48 h. The IL-6, IL-8, and VEGF mRNA and protein levels were determined by qRT-PCR or ELISA, respectively. **(A)** IL-6, IL-8, and VEGF mRNA levels in A549/H292 cells mRNA expression levels were calculated using the 2^−ΔΔCt^ method, and target gene expression was normalized to the GAPDH housekeeping gene. The data are presented as the mean ± SEM of three independent experiments. **(B)** IL-6, IL-8, and VEGF protein levels in A549/H292 cells by ELISA. The results shown are representative of four independent experiments and are presented the mean ± SEM. Comparisons were performed using the t-test. *p < 0.05; **p < 0.01; and ***p < 0.001.

To determine the role of IL-17 in IL-6, IL-8, and VEGF expression *in vitro*, A549 and H292 cells were pretreated with IL-17 (100 ng/ml) for 6 h, and then the IL-6, IL-8 and VEGF mRNA and protein levels were measured by qRT-PCR and ELISA. As expected, the IL-6, IL-8 and VEGF mRNA levels were increased by 8.2-, 3.6-, and 3.9-fold in A549 cells following treatment with IL-17 compared to treatment with medium alone (Fig. 4A). These results were further confirmed by ELISA (Fig. 4B). Similar increases were observed *in vitro* in H292 cells pretreated with IL-17 compared with medium. Taken together, these data indicate that IL-17 may promote IL-6, IL-8 and VEGF production in human lung adenocarcinoma *in vitro*.

### IL-17 activates STAT1 phosphorylation in lung adenocarcinoma cells *in vitro*

**Figure 5:**
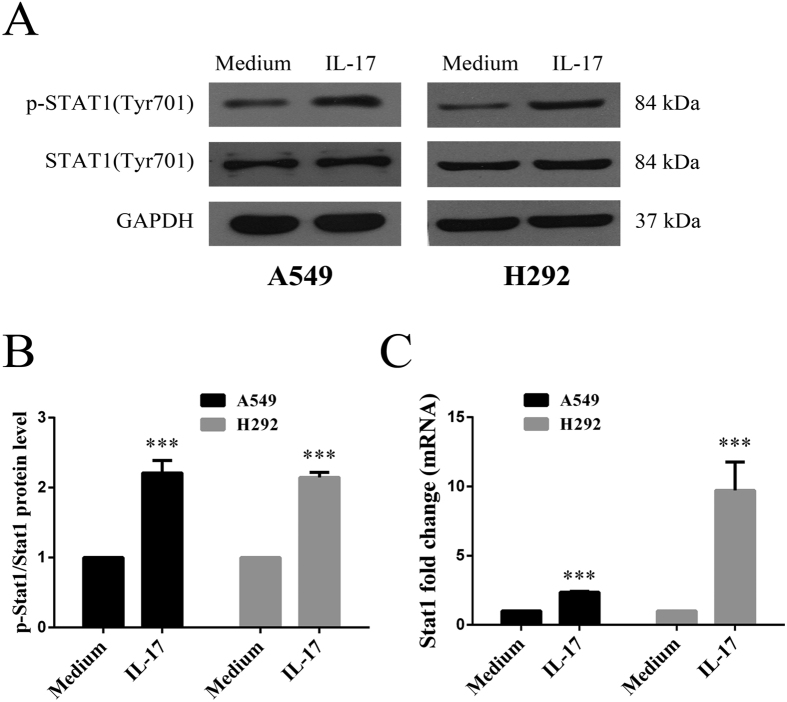
The effect of IL-17 on STAT1 signalling in human lung adenocarcinoma cells *in vitro*. **(A**,**B)** A549/H292 cells cells (1 × 10^6^ cells/well) were treated with or without 100 ng/ml IL-17 for 6 h, and then STAT1 protein levels were determined by WB. Target gene expression was normalized to the Gapdh housekeeping gene, and the data from three independent experiments are presented. **(C)** A549/H292 cells cells (5 × 10^5^ cells/well) were treated with or without 100 ng/ml IL-17 for 6 h, and then STAT1 mRNA levels were determined by qRT-PCR. mRNA expression levels were calculated using the 2^−ΔΔCt^ method, and target gene expression was normalized to the GAPDH housekeeping gene. The results shown are representative of four independent experiments and are presented the mean ± SEM. *p < 0.05 was regarded as significant. *Means p < 0.05, **means p < 0.01, ***means p < 0.001.

The biological effects of IL-17 have been shown to involve the Jak-Stat family. Thus, we further explored the possibility that IL-17 might modulate STAT1 phosphorylation in human lung adenocarcinoma cells. We determined the role of IL-17 in STAT1 phosphorylation in human lung adenocarcinoma cells *in vitro* by qRT-PCR and WB. As observed, STAT1 phosphorylation was increased in A549 cells following treatment with IL-17 compared to treatment with medium alone (Fig. 5A,B) by WB. Similar results were observed in H292 cells. And these data were proved at mRNA level in A549 and H292 (Fig. 5C). These results suggest that IL-17 activates STAT1 signalling in human lung adenocarcinoma cells *in vitro*.

### STAT1 knockdown attenuates IL-17-induced IL-6, IL-8, and VEGF expression in lung adenocarcinoma cells *in vitro*

**Figure 6:**
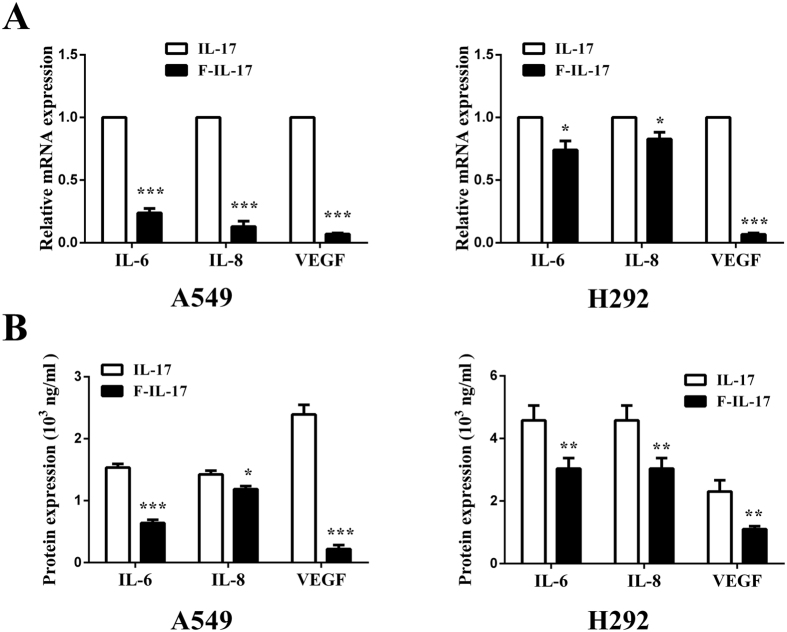
STAT1 inhibitor attenuated IL-17-induced IL-6, IL-8, and VEGF production in human lung adenocarcinoma. A549/H292 cells were incubated with IL-17 or IL-17 plus a STAT1 inhibitor for 6 or 48 h (100 ng/ml IL-17; and 30 μM inhibitor). The IL-6, IL-8, and VEGF mRNA and protein levels were determined by qRT-PCR and ELISA, respectively. **(A)** IL-6, IL-8, and VEGF mRNA levels in A549/H292 cells, mRNA expression levels were calculated using the 2^−ΔΔCt^ method, and target gene expression was normalized to the GAPDH housekeeping gene. The data are presented as the mean ± SEM of three independent experiments. **(B)** IL-6, IL-8, and VEGF protein levels in A549/H292 cells. The data are presented as the mean ± SEM of three independent experiments. Comparisons were performed using the t-test. *p < 0.05; **p < 0.01; and ***p < 0.001.

**Figure 7:**
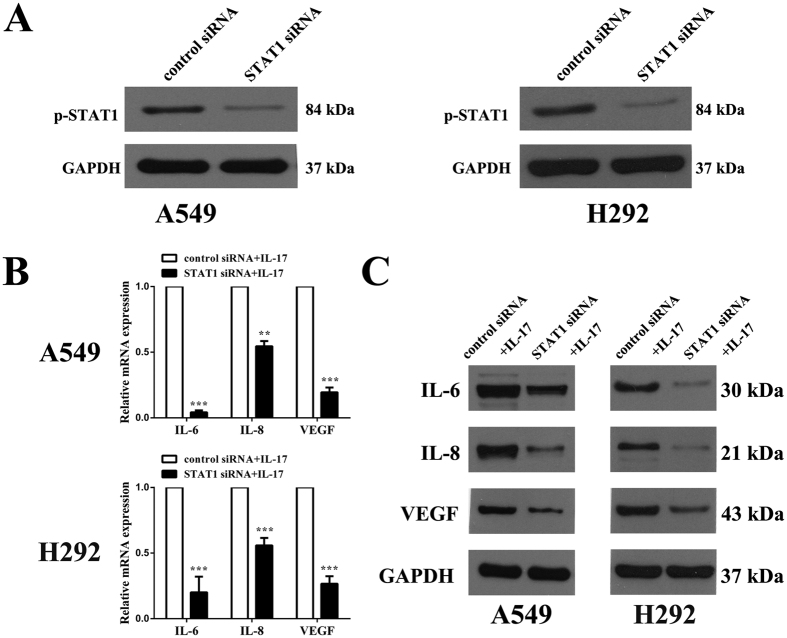
STAT1 siRNA reversed IL-17-induced IL-6, IL-8, and VEGF expression in human lung adenocarcinoma cells *in vitro*. A549/H292 cells (2 × 10^5^ cells/well) were transfected with STAT1 siRNA for 48 h. **(A)** STAT1 protein expression was determined by WB. Then, A549/H292 cells (5 × 10^5^ cells/well) were transfected with siRNA for 48 h and subsequently treated with human IL-17 (100 ng/ml) for an additional 6 or 48 h. The IL-6, IL-8, and VEGF mRNA and protein expression levels were determined by qRT-PCR and WB, respectively. **(B)** IL-6, IL-8, VEGF mRNA levels in A549/H292 cells. mRNA expression levels were calculated using the 2^−ΔΔCt^ method, and target gene expression was normalized to the GAPDH housekeeping gene. The data are presented as the mean ± SEM of three independent experiments. **(C)** IL-6, IL-8, and VEGF protein levels in A549/H292 cells and the results are presented as the mean ± SEM of three independent experiments. Comparisons were performed using the t-test. *p < 0.05; **p < 0.01; and ***p < 0.001.

To examine the effects of STAT1 on IL-17-induced IL-6, IL-8 and VEGF expression in A549 and H292 cells, a STAT1 inhibitor, fludarabine, and STAT1 siRNA were used to selectively inhibit STAT1 signalling. Our data suggested that fludarabine inhibits IL-17-induced IL-6, IL-8 and VEGF mRNA expression in both A549 and H292 cells (Fig. 6A). These findings were further confirmed by ELISA (Fig. 6B). In addition, transfection of A549 or H292 cells with STAT1 siRNA for 48 h resulted in effective STAT1 knockdown and a marked reduction in its activation, as determined by qRT-PCR and WB (Fig. 7A,B). The effects of STAT1 siRNA on A549 cells were similar to those of fludarabine and considerably attenuated the IL-17-mediated increases in IL-6, IL-8 and VEGF mRNA and protein expression compared to control siRNA, as determined by qRT-PCR and WB (Fig. 7C,D). Similar results were obtained in H292 cells. These findings suggest that STAT1 inhibits the IL-17-mediated elevations in IL-6, IL-8 and VEGF production in lung adenocarcinoma *in vitro*.

### IL-17 induces MVD in tumour tissues of A549-IL-17 cell-bearing nude mice

**Figure 8:**
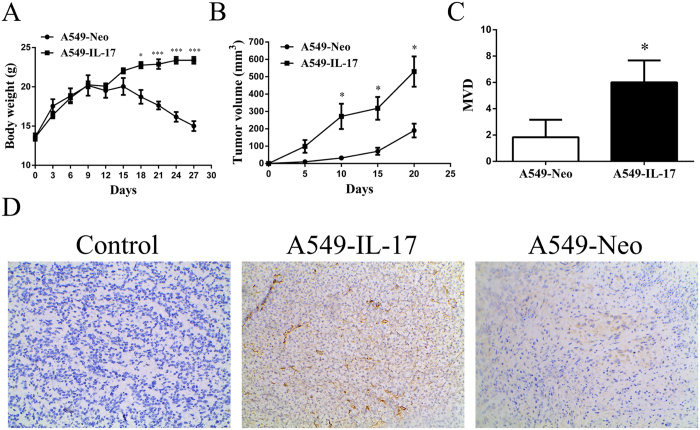
CD31 expression was increased in A549-IL-17 cell-bearing nude mouse tissues. **(A)** The time course of *in vivo* growth of A549-IL-17 cells vs. A549-Neo cells in nude mice (n = 5). **(B)** The time course of increases in the tumour volumes in A549-IL-17 and A549-Neo cells in nude mice (n = 5)**. (C)** The t-test was used to analyse the tumour microvessel densities in A549-IL-17 and A549-Neo cell-bearing nude mice (n = 5). **(D)** CD31 staining in tumour tissues from one each of A549-IL-17 and A549-Neo cell-bearing nude mice. For the control, one nude mouse tissue sample was not treated with a primary antibody. The data are presented as the mean ± SD for five mice per group, and the results are representative of two independent experiments. *p < 0.05; **p < 0.01; and ***p < 0.001.

Next, we generated A549 cells over-expressing the human IL-17 gene (A549-IL-17) to evaluate tumour growth and MVD in A549-IL-17 cell-bearing nude mice. Both A549-IL-17 and A549-Neo cells formed solid tumours when implanted s.c. in nude mice. A549-Neo cell-bearing nude mice displayed increased growth compared with controls (549-IL-17 cell-bearing nude mice) (body weight, p < 0.00; and TV, p < 0.05; Fig. 8A,B), suggesting that IL-17 may support tumour growth *in vivo*. Then, we determined if IL-17 increases MVD *in vivo* by performing CD31 staining in these mice. As expected, higher tumour vascularity was observed in the tumour tissues of the A549-IL-17 cell-bearing nude mice compared with the controls (Fig. 8C,D). These data provide evidence that IL-17 may promote tumor vascularity *in vivo*.

### IL-17 promotes IL-6, IL-8, VEGF and STAT1 phosphorylation in A549-IL-17 cell-bearing nude mice *in vivo*

**Figure 9:**
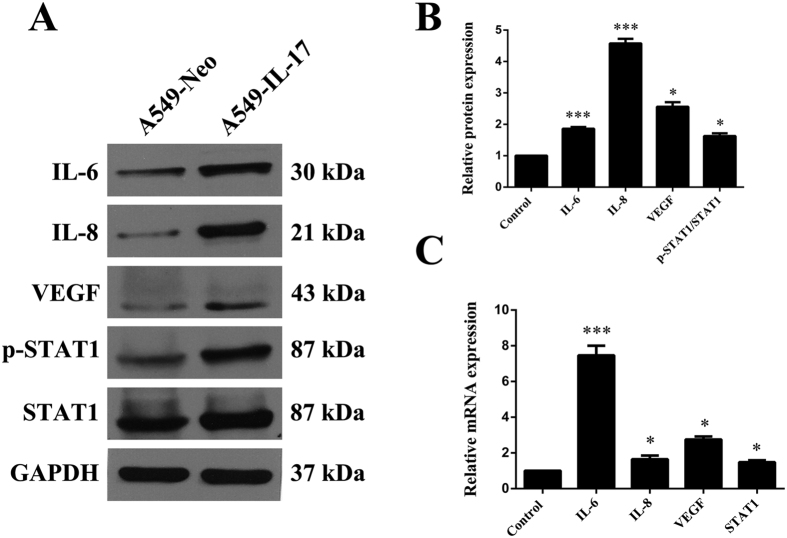
IL-6, IL-8, VEGF and STAT1 expression were augmented in A549-IL-17 cell-bearing nude mouse tissues. **(A**,**B)** The relative protein expression of IL-6, IL-8, VEGF and STAT1 in tumour tissues of A549-IL-17 vs. A549-Neo cell-bearing nude mice was determined by WB. **(C)** The relative mRNA expression of IL-6, IL-8, VEGF and STAT1 in tumour tissues of A549-IL-17 vs. A549-Neo cell-bearing nude mice was determined by qRT-PCR (n = 5). mRNA expression levels were calculated using the 2^−ΔΔCt^ method, and target gene expression was normalized to the GAPDH housekeeping gene. The data are presented as the mean ± SEM for five mice per group, and the results are representative of two independent experiments. *p < 0.05; **p < 0.01; and ***p < 0.001.

Given the correlation between IL-17 and MVD *in vivo*, we further assessed IL-6, IL-8 and VEGF production in A549-IL-17 cell-bearing nude mice by qRT-PCR and WB. IL-6, IL-8 and VEGF mRNA levels were increased by 2.3-, 4.1-, and 1.3-fold, respectively, in the tumour tissues of the A549-IL-17 cell-bearing nude mice compared with the mRNA levels in the controls (Fig. 9A). Similar changes in the protein levels were also observed (Fig. 9B). These results indicate that IL-17 promotes IL-6, IL-8 and VEGF expression in IL-17-overexpressing nude mice. The biological effects of IL-17 have been shown to involve the Jak-Stat family. In the present study, STAT1 phosphorylation in A549-IL-17 cell-bearing nude mice was measured by qRT-PCR and WB. STAT1 expression was slightly increased in the A549-IL-17 cell-bearing nude mice compared with controls (Fig. 9A,B), implying that IL-17 influences Stat1 expression *in vivo*.

## Discussion

In our study, MVD evaluated by CD31 staining was correlated with IL-17 expression in human lung adenocarcinoma tissues, as determined by qRT-PCR and IHC. Similarly, previous studies have confirmed that high IL-17 expression is significantly associated with high MVD by CD31 or CD34 staining in many types of tumours, such as human ovarian cancer^24^,^25^, hepatocellular carcinoma^26^, multiple myeloma^27^, colorectal carcinoma^28^, cholangiocarcinoma tumours^29^ and NSCLC^7^. Moreover, our CD31 staining results suggest that IL-17 increased MVD in A549-IL-17 cell-bearing nude mice. These findings are in agreement with a previous study^7^ demonstrating the presence of remarkably higher MVD by CD31 staining in tumour tissues of Sq-19-IL-17 cell-bearing SCID mice compared with Sq-19-Neo cell-bearing mice. Therefore, we conclude that IL-17 may promote tumour vascularity in lung adenocarcinoma. In addition, we have also revealed that IL-6, IL-8, and VEGF are positively associated with MVD by CD31 staining in human adenocarcinoma tissues, consistent with the results of previous studies that demonstrated that IL-6^30^, IL-8^31^ and VEGF^32^ are associated with angiogenesis in cancer, including NSCLC.

Given the proangiogenic property of IL-17, we further explored the effects of IL-17 on IL-6, IL-8 and VEGF expression. Our results suggested that IL-6, IL-8 and VEGF expression was positively correlated with IL-17 expression in human lung adenocarcinoma tissues. Additionally, IL-17 promoted IL-6, IL-8 and VEGF production in the A549-IL-17 cell-bearing nude mice. Our data showing that IL-17 stimulated IL-6, IL-8 and VEGF expression in the A549 cell line are in accordance with numerous studies reporting that IL-17 augments IL-6^20^,^33^,^34^, IL-8 and VEGF release^35^ in various types of non-tumour and tumour cells. With regard to lung cancer, IL-17 has been reported to induce IL-6 expression^13^ in A549 cells, and Numasaki M *et al*.^7^ have demonstrated that IL-17 increases IL-6 and IL-8 expression in A549, Sq-19 and LK-87 cells but that VEGF production is not altered in these three cell lines using ELISA. In contrast, Li Q *et al*.^15^ have reported that IL-17 increases VEGF expression in A549 cells using WB, and Li Y *et al*.^35^ have also found that IL-17 also promotes VEGF release from 95C and 95D cells using ELISA. The discrepancies regarding VEGF production among these previous studies may be partly attributed to differences in laboratory conditions or to the varying sensitivities of the different ELISA kits used. However, the mechanisms of the effects of IL-17 on IL-6, IL-8 and VEGF expression in the A549 cell line have not been previously elucidated.

Furthermore, we found that the STAT1 signalling pathway was involved in the IL-17-mediated induction of IL-6, IL-8 and VEGF expression in the A549 cells. In addition, we observed increased phosphorylation of p-STAT1 signalling in A549-IL-17 cell-bearing nude mice and in A549 cells following IL-17 stimulation. Accumulating evidence indicates that IL-17 directly communicates with the Jak/Stat and PI3K/Akt signalling pathways and that it also targets NF-kB, AP-1 and Sp1 in many tumour types^12^,^36^. For example, IL-17 has been reported to directly activate the tyrosine phosphorylation of STAT1, STAT2, STAT3 and STAT4 in human U937 monocytic leukaemia cells^37^ and of STAT1 and Stat3 in HaCaT cells^38^. In addition, previous studies have shown that Stat3/Akt regulates IL-6 production^20^, Akt modifies IL-8^39^ release, and Stat3/Akt modulate VEGF expression in lung cancer cell lines^39^,^40^. We therefore examined whether Stat1 participates in IL-17-induced angiogenic factor production in lung adenocarcinoma. As expected, STAT1 knockdown partially inhibited IL-17-mediated IL-6, IL-8, and VEGF production in human lung adenocarcinoma cell lines *in vitro*, suggesting communication between STAT1 signalling and IL-17 in lung adenocarcinoma.

In conclusion, we have revealed that IL-17 promotes IL-6, IL-8, and VEGF production in lung adenocarcinoma via STAT1 signalling. IL-6^41^, IL-8^42^, and VEGF^43^ are multifunctional cytokines that are regarded as biomarkers and are strongly associated with multiple aspects of lung cancer. Given the crosstalk that occurs between proinflammatory signalling pathways and IL-6, IL-8, and VEGF, as well as the pivotal roles of angiogenic factors in lung cancer, our work may facilitate the design of therapeutic interventions targeting both inflammation and angiogenesis in lung cancer in the future.

## Additional Information

**How to cite this article**: Huang, Q. *et al*. IL-17 Promotes Angiogenic Factors IL-6, IL-8, and Vegf Production via Stat1 in Lung Adenocarcinoma. *Sci. Rep*. **6**, 36551; doi: 10.1038/srep36551 (2016).

**Publisher’s note:** Springer Nature remains neutral with regard to jurisdictional claims in published maps and institutional affiliations.

## Supplementary Material

Supplementary Information
