# Corrigendum: IL-17 Promotes Angiogenic Factors IL-6, IL-8, and Vegf Production via Stat1 in Lung Adenocarcinoma

**DOI:** 10.1038/srep39566

**Published:** 2017-01-11

**Authors:** Qi Huang, Iimin Duan, Xin Qian, Jinshuo Fan, Zhilei Lv, Xiuxiu Zhang, Jieli Han, Feng Wu, Mengfei Guo, Guorong Hu, Jiao Du, Caiyun Chen, Yang Jin

Scientific Reports 6: Article number: 36551; 10.1038/srep36551 published online: 11 07 2016 updated: 01 11 2017.

This Article contains errors in the affiliations for Xin Qian, Jiao Du and Caiyun Chen, who were incorrectly listed as affiliated to “Department of Respiratory Medicine, Taihe Hospital, Hubei University of Medicine, No. 32, South Renmin Road, Shiyan, Hubei, 442000, P.R. China”, “Zhongshan Hospital, Xiamen University, 201-209 Hubin Road, Xiamen, Fujian, 361004, P.R. China” and “Department of Respiratory Medicine,the First Hospital of Xi′an City, Xi′an, Shanxi, 710002, P.R. China” respectively.

The correct affiliation for all authors is given below:

Department of Respiratory and Critical Care Medicine, Key Laboratory of Pulmonary Diseases of Health Ministry, Union Hospital, Tongji Medical College, Huazhong University of Science and Technology, 1277 Jiefang Avenue, Wuhan, 430022, China.

