# Supplementary material for: IL-17 Promotes Angiogenic Factors IL-6, IL-8, and Vegf Production via Stat1 in Lung Adenocarcinoma
Source: Sci Rep. 2016 Nov 7;6:36551. doi: 10.1038/srep36551 (PMC5098156; doi:10.1038/srep36551)
Supplement: Supplementary Information [file srep36551-s1.docx]

**IL-17 promotes angiogenic factors IL-6, IL-8, AND VEGF PRODUCTION** **VIA STAT1 IN LUNG ADENOCARCINOMA**

**Running title:** IL-17-mediated IL-6, IL-8, and VEGF production via STAT1

Qi Huang^1^, limin Duan^1#^, Xin Qian^2#^, Jinshuo Fan^1#,^ Zhilei Lv^1^,Xiuxiu Zhang^1^, Jieli Han^1^, Feng Wu^1^,Mengfei Guo^1^, Guorong Hu^1^, Jiao Du^3^, Caiyun Chen^4^, Yang Jin^1*^

1. Department of Respiratory and Critical Care Medicine, Key Laboratory of Pulmonary Diseases of Health Ministry, Union Hospital, Tongji Medical College, Huazhong University of Science and Technology, 1277 Jiefang Avenue, Wuhan, Hubei,430022, P.R. China
2. Department of Respiratory Medicine, Taihe Hospital, Hubei University of Medicine, No. 32, South Renmin Road, Shiyan, Hubei, 442000, P.R. China.
3. Zhongshan Hospital, Xiamen University, 201-209 Hubin Road, Xiamen, Fujian, 361004, P.R. China.
4. Department of Respiratory Medicine,the First Hospital of Xi′an City, Xi′an, Shanxi, 710002, P.R. China.

**Supplementary figure 1**

The expression of IL-17 protein and mRNA in A549 (A549-IL-17/A549-Neo) was verified by immunofluorescence and real time PCR. (A) one clone of A549-Neo cells. (B,C,D)three clones of A549-IL-17 cells. (E) IL-17 mRNA expression in three clones of A549-IL-17 cells compared to A549-Neo cells.


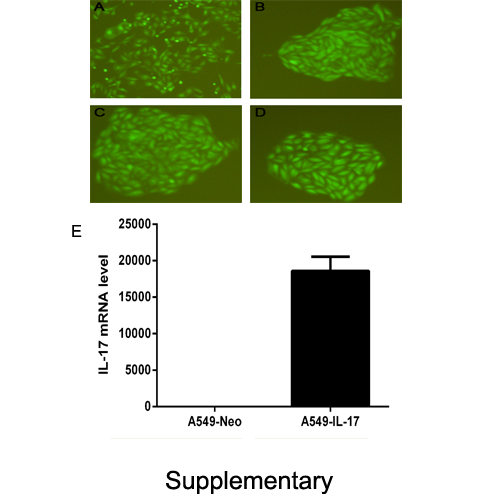


**Supplementary table 1. Sequence of primers used for qRT-PCR amplification**

| **Gene** | **Forward primer(5′-3′)** | **Reverse primer(5′-3′)** |
| --- | --- | --- |
| **Human gene** |  |  |
| **IL-17A**  **IL-6**  **IL-8**  **VEGF**  **Stat1** | **ACCTGAACATCCATAACCGGAATAC**  **AAGCCAGAGCTGTGCAGATGAGTA**  **ACACTGCGCCAACACAGAAATTA**  **GAGCCTTGCCTTGCTGCTCTAC**  **ATCACATTCACATGGGTGG** | **AGCGTTGATGCAGCCCAAG**  **TGTCCTGCAGCCACTGGTTC3**  **TTTGCTTGAAGTTTCACTGGCATC**  **CACCAGGGTCTCGATTGGATG**  **CTTCAGGGGATTCTCAGGAATA** |
| **GAPDH** | **GCACCGTCAAGGCTGAGAAC** | **TGGTGAAGACGCCAGTGGA** |
|  |  |  |
